# The Strength of Metropolitan Voluntary Hospitals

**Published:** 1914-05-09

**Authors:** 


### The Hospital

**The Workers' Newspaper of Administrative Medicine and Institutional Life, Administration, National Insurance and Health.**

No. 1454, Vol. LVI. SATURDAY, MAY 9, 1914. PRICE ONE PENNY.

### THE STRENGTH OF METROPOLITAN VOLUNTARY HOSPITALS.

It is but seventeen years since the King's Hospital Fund was established with the object of stiffening and improving the management, development, and equipment of the London voluntary hospitals as a whole, and of securing for them adequate financial support, with the enforcement of economical administration upon identical lines. The Report of the King's Hospital Fund for 1913, which was adopted at the annual meeting at St. James's Palace on the 30th ultimo, is interesting reading from every point of view. In the seventeen years upwards of one and three-quarter million pounds sterling have been raised and distributed amongst the hospitals, convalescent homes, and consumption sanatoria. The investments of the Fund at the present time exceed two million pounds sterling. The League of Mercy, which has been responsible for raising subscriptions in small sums from 1s. a year upwards, has since 1898 handed to the King's Fund £202,000, apart from the sums it has distributed to hospitals ministering where the League works to the population of the home and other counties which are outside the King's Fund's area.

The organisation of the work of the King's Fund, its business and financial management, and the invaluable voluntary service it has secured, on behalf of the hospitals of London, from the leading men of the day, representing the wide field of scientific, medical, architectural, financial, economical, nursing, and hygienic achievement covered by the hospitals of the Metropolis, bear testimony to the wisdom and statesmanlike qualities of King Edward and his advisers, to whom the country is indeed indebted for this, the finest and most valuable, system of hospital organisation the world has yet seen. From the obscurest patient to the greatest leaders of religious thought and development; from the trained nurse to the medical and surgical experts who are to-day leaders of the great profession of medicine; from the humblest worker in the field of charity to the greatest financiers and foremost men of business, the King's Hospital Fund for London has done good service, and has so come to be welcomed and held in high honour by the English people. King Edward's example as a pioneer in personal service, and the convinced upholder and supporter of our voluntary hospitals, has been followed with equal conviction and equal helpfulness to the sick by our present King George, and by the members of His family. Men of many professions, leaders of thought and influence in all fields of active life have gladly given voluntary service in the cause of the hospitals as members of the Council and committees and as visitors of the hospitals and convalescent homes which are covered by the activities of this great Fund. We have recently been going over the work and organisation during the seventeen years of its existence, and have closely examined into the condition and position of the Fund to-day. We make bold to say that there is nothing in the whole field of charity to-day, nor even in the business world, which can better stand the tests which collectively prove the greatness of this work, and show that it is good from end to end.

We have recently dealt with the subject of legacies, and venture to lay down that those institutions which do not maintain an average contribution from this source must in justice attribute the diminished supply to faulty methods, or a want of up-to-date, competent administration and adequate driving force. A great voluntary hospital or kindred institution cannot in the present day thrive and extend its work, unless it is blessed by the service of at least one man or woman, or both, of marked ability and commendable energy who continuously give of themselves in furtherance of the work. The latest and one of the most remarkable examples of the truth of this contention is Sir J Wolfe-Barry in his action in connection with the Westminster Hospital, which has to face the responsibility of finding a new site, creating a modern, up-to-date hospital, with a perfected medical school equipment, and to take its share, and a large share, of the medical and surgical work engendered by the needs of the population of London. The King's Fund Report calls attention once more to the magnificent legacy, being one-twelfth of his whole fortune, left to that Fund by the late Sir Julius Wernher, one of the original members of its Council and one of the ablest and most successful of business men. We welcome the marked and emphatic expressions of gratitude and appreciation of this noble gift, to be found in the gracious message read from the King, in the Report of the King's Fund, and in the graceful and eloquent testimony borne by the Duke of Teck in his speech as chairman of the meeting. We often get complaints that legacies are fewer than formerly. We should like to see the example of the King's Fund's generous appreciation followed by all institutions in regard to those good friends who, on leaving the world, do not fail to leave behind them substantial testimony to their interest and belief in the charities with which during life they may have been associated.

The great body of citizens throughout London, or the more thoughtful and best of them, owe it to themselves to make it their business, following the example of the King and Queen and their children, to send to the King's Fund every year a small subscription from their family circle, even if it does not exceed in the whole one guinea per household. The splendid work and enormous good effected by the King's Hospital Fund should receive general recognition by a multitude of small gifts from Londoners, resident or working within the Metropolis of the Empire. It is the spontaneity of gifts like these which the Fund deserves. The receipt of such gifts, too, quite apart from their amount, would be invaluable as an encouragement to the many workers by whose collective energies the splendid results here recorded have been made possible.

